# Dual p38/JNK Mitogen Activated Protein Kinase Inhibitors Prevent Ozone-Induced Airway Hyperreactivity in Guinea Pigs

**DOI:** 10.1371/journal.pone.0075351

**Published:** 2013-09-18

**Authors:** Kirsten C. Verhein, Francesco G. Salituro, Mark W. Ledeboer, Allison D. Fryer, David B. Jacoby

**Affiliations:** 1 Department of Physiology and Pharmacology, Oregon Health and Science University, Portland, Oregon, United States of America; 2 Division of Pulmonary and Critical Care Medicine, Oregon Health and Science University, Portland, Oregon, United States of America; 3 Vertex Pharmaceuticals, Inc., Cambridge, Massachusetts, United States of America; Research Center Borstel, Germany

## Abstract

Ozone exposure causes airway hyperreactivity and increases hospitalizations resulting from pulmonary complications. Ozone reacts with the epithelial lining fluid and airway epithelium to produce reactive oxygen species and lipid peroxidation products, which then activate cell signaling pathways, including the mitogen activated protein kinase (MAPK) pathway. Both p38 and c-Jun NH_2_ terminal kinase (JNK) are MAPK family members that are activated by cellular stress and inflammation. To test the contribution of both p38 and JNK MAPK to ozone-induced airway hyperreactivity, guinea pigs were pretreated with dual p38 and JNK MAPK inhibitors (30 mg/kg, ip) 60 minutes before exposure to 2 ppm ozone or filtered air for 4 hours. One day later airway reactivity was measured in anesthetized animals. Ozone caused airway hyperreactivity one day post-exposure, and blocking p38 and JNK MAPK completely prevented ozone-induced airway hyperreactivity. Blocking p38 and JNK MAPK also suppressed parasympathetic nerve activity in air exposed animals, suggesting p38 and JNK MAPK contribute to acetylcholine release by airway parasympathetic nerves. Ozone inhibited neuronal M_2_ muscarinic receptors and blocking both p38 and JNK prevented M_2_ receptor dysfunction. Neutrophil influx into bronchoalveolar lavage was not affected by MAPK inhibitors. Thus p38 and JNK MAPK mediate ozone-induced airway hyperreactivity through multiple mechanisms including prevention of neuronal M_2_ receptor dysfunction.

## Introduction

Over half the United States population lives in counties with unhealthy levels of ozone, a major component of smog [1]. Epidemiological studies demonstrate a significant link between exposure to ground level ozone and pulmonary hospitalizations. Exposure to ozone in excess of 0.16 ppm is associated with increased airway reactivity, lung inflammation and exacerbation of asthma in both adults and children [2], [3], [4].

Ozone induced hyperreactivity is demonstrated by increased reactivity to inhaled methacholine and other agonists, including those causing reflex bronchoconstriction in man [5], [6], [7]. In animals, ozone induced airway hyperreactivity is demonstrated by increased bronchoconstriction to intravenous methacholine, but this effect is mediated largely via increased acetylcholine release from parasympathetic nerves, since it is blocked by vagal section [8], [9]. Direct stimulation of the vagus nerves results in bronchoconstriction that is potentiated in ozone exposed animals and that is associated with loss of function of neural M_2_ muscarinic receptors that normally inhibit acetylcholine release [10], [11]. Inflammatory cells, especially eosinophils through release of the M_2_ inhibitor major basic protein, mediate loss of neuronal M_2_ function and airway hyperreactivity in ozone exposed guinea pigs [11].

However, ozone is unlikely to contact inflammatory cells [12]. At the airway epithelial layer, ozone forms reactive oxygen species and lipid peroxides in lungs of humans and animals [13], [14]. These end products activate cell signaling pathways, including mitogen activated protein kinase pathways (MAPK) [15]. Activation of the MAPK pathway results in inflammation [16], mucus hypersecretion [17] and airway hyperreactivity [18].

MAPK signaling pathways are important in many cell processes including differentiation, proliferation, activation, degranulation, and migration. Three MAPK subfamilies have been well characterized: ERK, JNK, and p38. The extracellular signal-regulated kinase (ERK) pathway is usually activated by mitogens and growth factors while p38 and c-Jun NH_2_ terminal kinase (JNK) pathways are associated with chronic inflammation and are typically activated by inflammatory cytokines, heat shock, and cellular stress [19], [20]. Activation of MAPK signaling induces inflammatory cytokine and chemokine production in airway epithelial cells, inflammatory cells, and airway smooth muscle cells [16], [21], [22]. Humans with severe asthma have increased activated p38 in airway epithelium compared to mild asthmatics or healthy controls, as demonstrated by increased immunostaining of phosphorylated p38 in airway biopsies [23].

Inhibition of MAPKs is protective in allergen challenge models of asthma. Inhibition of p38, either pharmacologically or with antisense oligonucleotides, partially prevents airway hyperreactivity after sensitization and challenge in mice [18], [24]. Eosinophil influx into bronchoalveolar lavage is the dominant event in antigen challenged animals, and is prevented by a p38 inhibitor in guinea pigs and mice [25]. Blocking p38 also prevents IL-13 induced mucus metaplasia in human and mouse airway epithelial cells [17], [26].

Less is known about the role of the MAP kinases in ozone-induced hyperreactivity. Inhibiting p38 prevents ozone-induced airway hyperreactivity in mice while inhibiting JNK is partially protective [27], [28]. Ozone-induced increases in inflammatory cells in bronchoalveolar lavage are significantly inhibited in *Jnk1* knockout mice [29].

The experiments described here use three different MAPK inhibitors to test whether dual inhibition of both p38 and JNK MAPK pathways prevents ozone-induced inflammation and subsequent airway hyperreactivity in guinea pigs.

## Methods

### Ethics Statement

Guinea pigs were handled in accordance with the standards established by the United States Animal Welfare Act set forth in National Institutes of Health guidelines. All protocols were approved by Oregon Health and Science University Animal Care and Use Committee (protocol A984).

### Animals

Specific pathogen-free female Hartley guinea pigs (300–470 g; Elm Hill Breeding Labs, Chelmsford, MA) were shipped in filtered crates, housed in high efficiency particulate filtered air, and fed a normal diet.

### Ozone Exposure

Guinea pigs were exposed to 2 ppm ozone or filtered air for 4 hours as described previously [11]. Physiological measurements, airway inflammation, and histological measurements were made one day after a single ozone exposure.

### Treatment of Guinea Pigs with p38 and JNK MAPK Inhibitors

**Figure 1:**
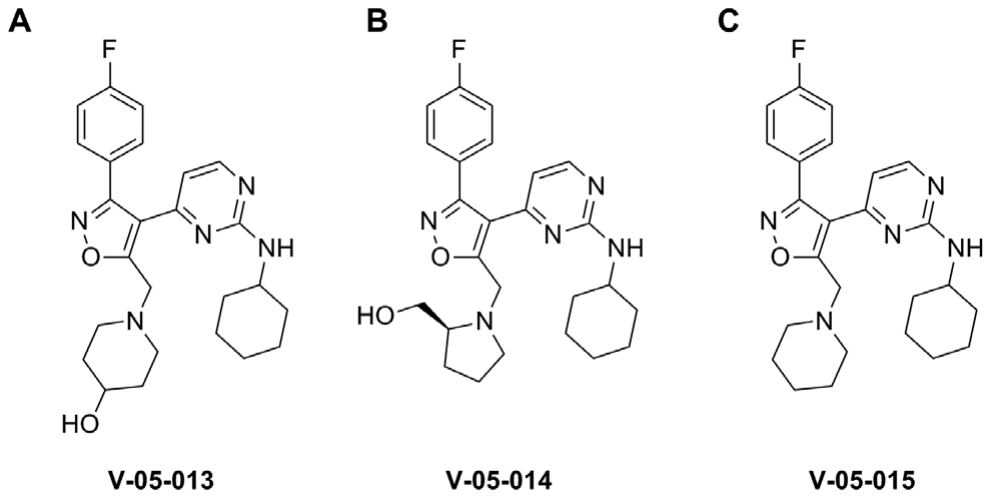
Chemical structures of dual p38 and JNK MAPK inhibitors.

Animals were given 30 mg/kg intraperitoneally of the dual p38 and JNK MAPK inhibitors V-05-013, V-05-014, or V-05-015 (Vertex Pharmaceuticals, Cambridge, MA) one hour before ozone exposure (Figure 1). These compounds were chosen because of their overall kinase selectivity profile. They are potent and selective dual inhibitors of p38 and JNK (see below and table 1) and they do not show activity against a panel of other kinases at concentrations <1 µM (see characterization data below). Inhibitors were dissolved in 25% DMSO in phosphate buffered saline (PBS). Air exposed control animals were given 25% DMSO in PBS one hour before ozone exposure.

**Table 1:** Ki values for dual p38 and JNK MAPK inhibitors.

Ki (nM)	V-05-013	V-05-014	V-05-015
p38	13	15	8*
JNK1	170	300	120
JNK2	10	15	4
JNK3	5	10	8

All compounds have a Ki greater than 1 µM for all other kinases tested.

* This one value is an IC50, not a Ki.

All three drugs have similar kinase inhibition profiles and exhibit potent affinity for both p38 and JNK. Affinity was measured using a kinase inhibition assay. Compounds were assayed for the inhibition of various kinases using a modification of a spectrophotometric coupled-enzyme assay [30]. In this assay, a fixed concentration of activated kinase (10–40 nM) was incubated with various concentrations of a potential inhibitor dissolved in DMSO for 10 minutes at 30°C in a buffer containing 0.1 M HEPES, pH 7.5, containing 10 mM MgCl_2_, 2.5 mM phosphoenolpyruvate, 200 µM NADH, 2 mM DTT, 30 µg/mL pyruvate kinase, 10 µg/mL lactate dehydrogenase, and 200 µM–500 µM EGF receptor peptide. The EGF receptor peptide has the sequence KRELVEPLTPSGEAPNQALLR. The reaction was initiated by the addition of ATP equal to the ATP Km of the kinase, and the assay plate is inserted into the spectrophotometer’s assay plate compartment that was maintained at 30°C. The decrease of absorbance at 340 nm was monitored as a function of time for 10 minutes. The rate data as a function of inhibitor concentration was either fit as an IC50 or to a competitive inhibition kinetic model to determine the compound K_i_ (see table 1).

Proton NMR spectra for the compounds was recorded on a Bruker Advance instrument with a QNP probe using TMS as the internal standard in the indicated deuterated solvent. LC−MS analyses were performed on a Waters ZQ or ZMD or QuatroII mass spectrometer using the electrospray (ESI) ionization technique. Samples were introduced into the mass spectrometer using chromatography. Methods (LC-MS) consisted of the following: 5–95% Water/Acetontrile (0.1% TFA) over 0.6 min on a Waters Acquity CSH C18, 1.7 µm, 2.1×50 mm column, with a flow rate of 0.6 mL/min. NMR spectra for each compound are below.

**V-05**-**013∶**1H NMR (300 MHz, MeOD) 8.20 (d, J = 5.6 Hz, 1H), 7.62 (dd, J = 5.3, 8.6 Hz, 1H), 7.29 (t, J = 8.6 Hz, 1H), 6.64-6.47 (m, 1H), 5.03 (s, H), 4.18-4.00 (m, 1H), 4.18-4.00 (m, 1H), 3.80-3.35 (m, 5H), 1.71 (m, 1H), 1.52-1.23 (m, 5H); LC-MS (method X) t_R_ = 0.60 min., (M+H^+^) 452.37. Ki or IC50 was determined to be >1 µM for the following kinases: AKT3, AurA, CDK2, ERK-2, EphA, FLT3, IGF1R, IRAK, ITK, JAK2, JAK3, KDR, MAPKAP2, cMET, MKK4, MKK6, MKK7, PIM1, PKA, PLK1, PRAK, ROCK1, SRC, SYK, TIE2, ZAP70; GSK3β Ki = 0.73 µM.

**V-05-014∶**1H NMR (400 MHz, DMSO-d6) 11.04 (s, 0.3 H), 10.22 (s, 0.2 H), 8.33 (s, 1 H), 8.08 (s, 1 H), 7.38–7.79 (m, 4 H), 6.68 (s, 1 H), 6.20 (s, 1 H), 5.15–5.23 (m, 0.7 H), 4.86–4.91 (m, 1H), 3.78 (s, 3 H), 3.53 (s, 1 H), 3.33 (s, 1 H), 1.56–2.13 (m, 9 H), 1.09–1.28 (m, 6 H); LC-MS (method Y) ^t^R = 2.47 min., (M+H^+^) 452.25; LC-MS (method X) t_R_ = 0.63 min., (M+H^+^) 452.37. Ki or IC50 was determined to be >1 µM for the following kinases: AKT3, AurA, COT, CDK2, ERK2, EphA, FAK, GSK3β, IGF1R, IRAK, JAK3, KDR, LCK, MAPKAP2, cMET, MKK4, MKK6, MKK7, NIK, PDK1, PIM1, PKA, PLK1, PRAK, ROCK1, SRC, SYK, TIE2, ZAP70.

**V-05-015∶**1H NMR (300 MHz, MeOD) 8.20 (d, J = 5.1 Hz, 1H), 7.62 (dd, J = 5.3, 8.6 Hz, 2H), 7.29 (t, J = 8.7 Hz, 2H), 6.68-6.42 (m, 1H), 5.00 (s, 2H), 3.75-3.53 (m, 3H), 3.34-3.15 (m, 2H), 2.10-1.77 (m, 9H), 1.88 (m, 1H), 1.65-1.20 (m, 7H); LC-MS (method X) t_R_ = 0.64 min., (M+H^+^) 436.38. Ki or IC50 was determined to be >1 µM for the following kinases: AKT3, AurA, CDK2, ERK2, FLT3, GSK3b, IGF1R, IRAK, JAK2, JAK3, KDR, LCK, MAPKAP2, cMET, MKK4, MKK6, MKK7, PDK1, PIM1, PKA, PLK1, PRAK, ROCK1, SRC, SYK, ZAP70.

### Measurement of Pulmonary Inflation Pressure

One day after exposure to ozone, guinea pigs were anesthetized with 1.9 g/kg urethane i.p. (Sigma-Aldrich, St. Louis, MO). This dose produces a deep anesthesia lasting 8–10 hours [31] though no experiments lasted longer than 4 hours.

Physiological measurements were made as previously described [32]. The jugular veins were cannulated for intravenous administration of drugs and the right carotid artery was cannulated to measure heart rate and blood pressure. Both vagus nerves were cut and distal ends placed on platinum electrodes submerged in liquid paraffin. Animals were tracheostomized, ventilated (1 ml/100 g body weight, 100 breaths per minute) and paralyzed with a constant infusion of succinylcholine (10 µg/kg/min iv, Sigma-Aldrich). Pulmonary inflation pressure was measured at the trachea and bronchoconstriction was measured as the increase in pressure over basal inflation pressure produced by the ventilator.

### Measurement of Vagally Induced Bronchoconstriction

Electrical stimulation of both vagus nerves (10V, 0.2 ms pulse width, 1–25 Hz, 5 sec duration at 1 minute intervals) produced frequency dependent bronchoconstriction and bradycardia due to release of acetylcholine onto muscarinic receptors. To confirm vagally induced bronchoconstriction was cholinergic, atropine (1 mg/kg iv, Sigma-Aldrich) was given at the end of each experiment.

### Measurement of Smooth Muscle M_3_ Muscarinic Receptor Function

Recovery from vagal stimulation was confirmed by pulmonary inflation pressure and heart rate returning to baseline before measuring smooth muscle M_3_ muscarinic receptor function (5–10 minutes after cessation of vagal stimulation). In vagotomized guinea pigs, M_3_ muscarinic receptor function on airway smooth muscle was tested by measuring bronchoconstriction after administration of acetylcholine (1–10 µg/kg iv, Sigma-Aldrich).

### Measurement of Neuronal M_2_ Muscarinic Receptor Function

Recovery from administration of intravenous acetylcholine was confirmed by pulmonary inflation pressure and heart rate returning to baseline before measuring M_2_ muscarinic receptor function (5–10 minutes after the last dose of acetylcholine). To test the function of neuronal M_2_ muscarinic receptors, vagally induced bronchoconstriction was measured before and after administration of gallamine (0.1–10 mg/kg iv, Sigma-Aldrich) an M_2_ receptor antagonist. Electrical stimulation of both vagus nerves (3–30V, 0.2 ms pulse width, 15 Hz, 5 sec duration at 1 minute intervals) produced reproducible, frequency dependent, bronchoconstrictions. In the presence of normally functioning M_2_ receptors, gallamine (0.1–10 mg/kg iv) will block them, resulting in increased vagally induced bronchoconstriction [33]; an effect that is suppressed if M_2_ receptors are not responding to endogenous acetylcholine [10].

### Bronchoalveolar Lavage (BAL)

At the end of each experiment, the lungs were lavaged with five 10 ml aliquots of phosphate buffered saline (PBS) that contained 100 µg isoproterenol (Sigma-Aldrich). Lavage fluid was centrifuged (400 *g*, 10 min) and the pellets were resuspended in PBS. Cells were counted using a hemocytometer and slides made from centrifuged lavaged cells were stained with Hemacolor (EMD Chemicals, Gibbstown, NJ) and used to determine cell differentials.

### Drugs

Acetylcholine, succinylcholine, and urethane were purchased from Sigma (St. Louis, MO) and were dissolved and diluted in PBS.

### Data Analysis and Statistics

All data are expressed as means ± SE. *In vivo* frequency response and dose response curves were compared using two-way ANOVA for repeated measures. Baseline data were analyzed by one-way ANOVA with Bonferroni’s correction. A *P* value of less than 0.05 was considered significant. Analyses were made with GraphPad Prism (version 5.0; GraphPad Software, La Jolla, CA).

## Results

### Baselines

One day after ozone exposure, baseline pulmonary inflation pressure was significantly increased compared to air-exposed controls (Table 2). All the dual p38 and JNK inhibitors partially attenuated the ozone induced increase in baseline airway inflation pressure, although the attenuation only reached statistical significance in the group treated with V-05-013. None of the MAPK inhibitors affected baseline inflation pressure in air-exposed controls. Neither ozone nor the MAPK inhibitors affected baseline heart rate or blood pressure.

**Table 2:** Baseline cardiovascular and pulmonary parameters.

		Heart Rate	Blood Pressure (mmHg)		Pulmonary Inflation
Group	*n*	(beats/min)	Systolic	Diastolic	Pressure (mmH_2_O)
Air	7	306±6	40±4	20±3	110±4
Ozone	5	309±10	48±4	23±4	248±21 *
Ozone+V-05-013	5	301±7	40±3	20±2	190±15 * ^‡^
Ozone+V-05-014	4	286±16	38±4	21±1	225±5 *
Ozone+V-05-015	3	353±7	40±1	18±1	213±7 *
Air+V-05-013	4	291±7	32±2	17±1	102±3
Air+V-05-014	5	306±9	38±2	19±1	102±2
Air+V-05-015	5	327±14	40±3	22±3	94±5

Values are means ± SEM. Baseline pulmonary inflation pressure significantly increased after ozone exposure. Treatment with dual p38/JNK MAPK inhibitor V-05-013 significantly reduced the ozone-induced increase in baseline pulmonary inflation pressure. *p<0.05 Significantly different from air exposure. ^‡^p<0.05 Significantly different from ozone exposure.

### Airway Physiology

**Figure 2:**
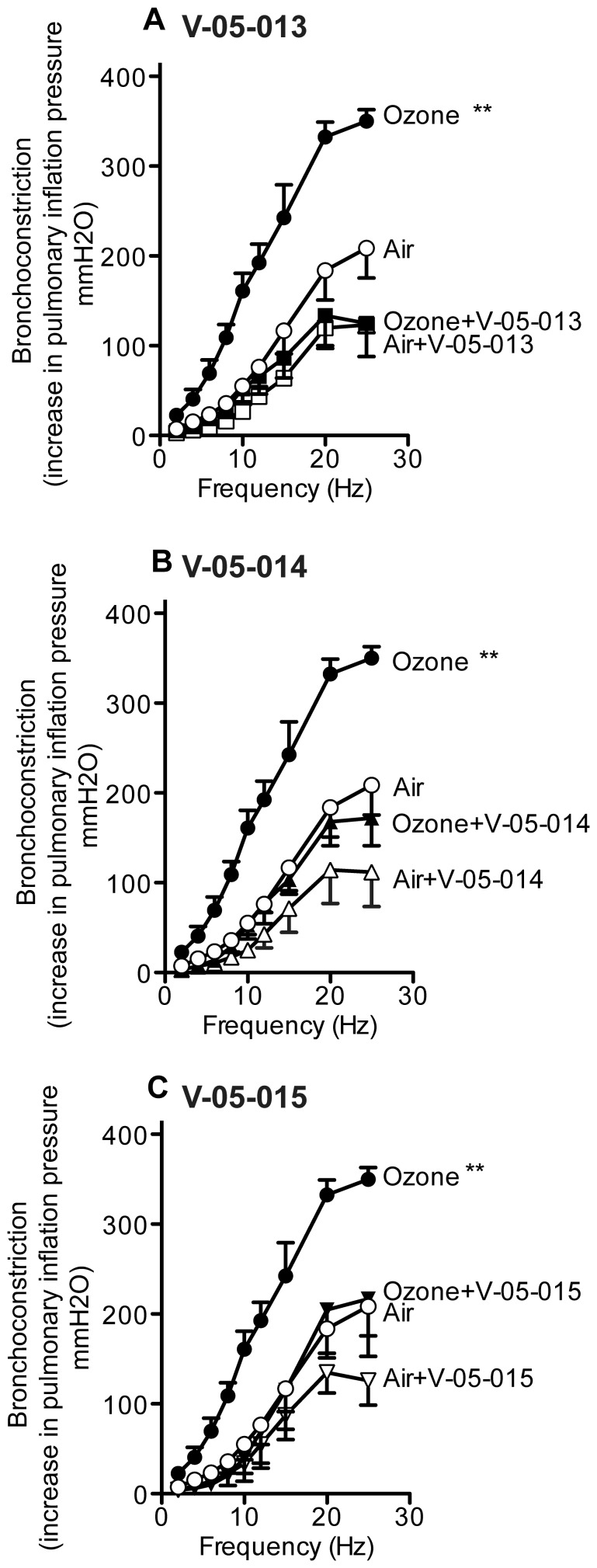
Blocking p38 and JNK MAPK completely prevented ozone-induced airway hyperreactivity mediated by the vagus nerves. In anesthetized and vagotomized guinea pigs, stimulation of the vagus nerves (10V, 0.2 ms pulse width, 1–25 Hz, 5 sec duration at 1 minute intervals) caused frequency dependent bronchoconstriction (A open circles; measured as an increase in inflation pressure in mmH_2_O) that is significantly potentiated one day post-ozone exposure (A closed circles). Pretreatment with dual MAPK inhibitors V-05-013 (A closed squares), V-05-014 (B closed triangles), or V-05-015 (C closed inverted triangles) completely prevented ozone-induced airway hyperreactivity. All three dual MAPK inhibitors suppressed parasympathetic nerve activity (A open squares, B open triangles, C open inverted triangles). Ozone and air exposed control data are the same in A-C. *p<0.05, **p<0.01 Significantly different from air exposed controls. Data are mean ± SEM. n = 4–7.

**Figure 3:**
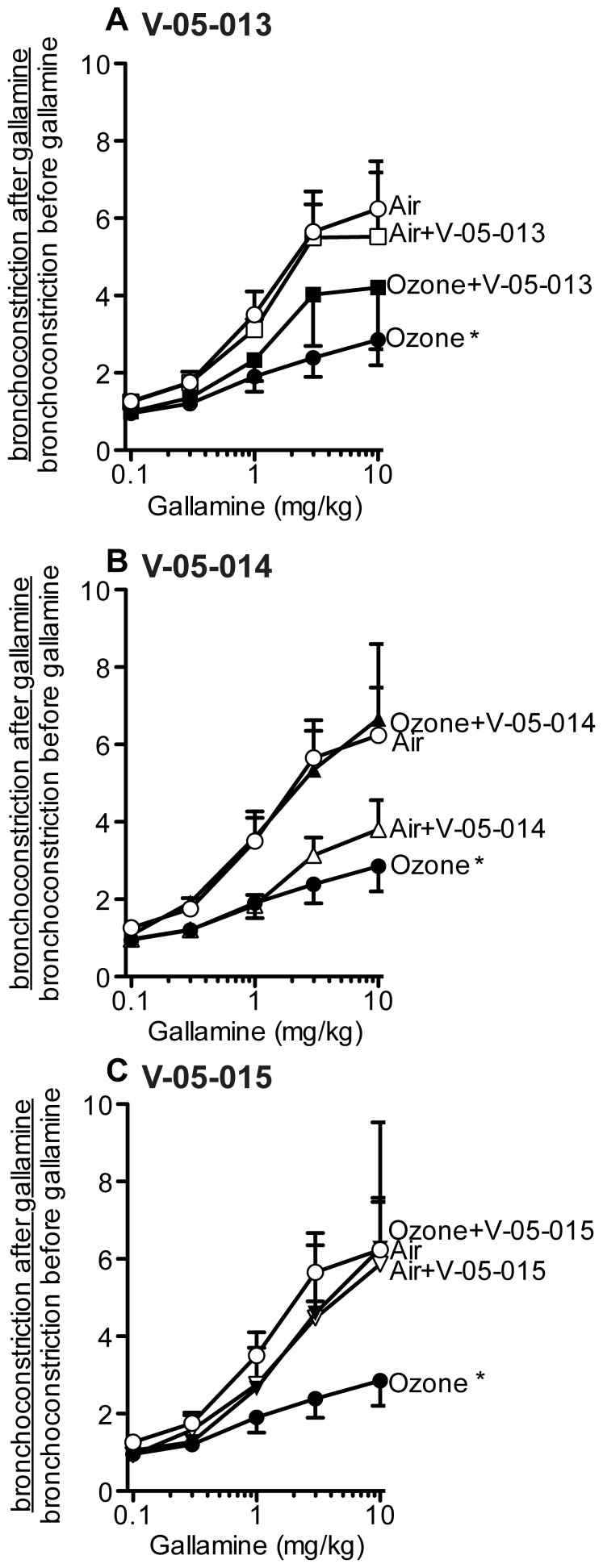
In control (air exposed) guinea pigs electrical stimulation of the vagus nerves (3–30V, 0.2 ms pulse width, 15 Hz, 5 sec duration at 1 minute intervals) resulted in vagally induced bronchoconstriction (measured as an increase in pulmonary inflation pressure; 16±1 mmH_2_O). An M_2_ receptor antagonist, gallamine, potentiated vagally induced bronchoconstriction up to 6-fold in air exposed animals (open circles) demonstrating that functional M_2_ receptors were limiting acetylcholine release. The potentiation by gallamine was decreased in ozone-exposed animals, demonstrating M_2_ receptors were dysfunctional after ozone exposure (closed circles). V-05-013 partially prevented M_2_ receptor dysfunction (C closed squares), while V-05-014 (B closed triangles) and V-05-015 (C closed inverted triangles) completely protected M_2_ receptor function. Vagally induced bronchoconstriction in the absence of gallamine was not different from control among all groups. Ozone and air exposed controls are the same in A–C. *p<0.05, **p<0.01 Significantly different from air exposed controls. Data are mean ± SEM. n = 4–7.

**Figure 4:**
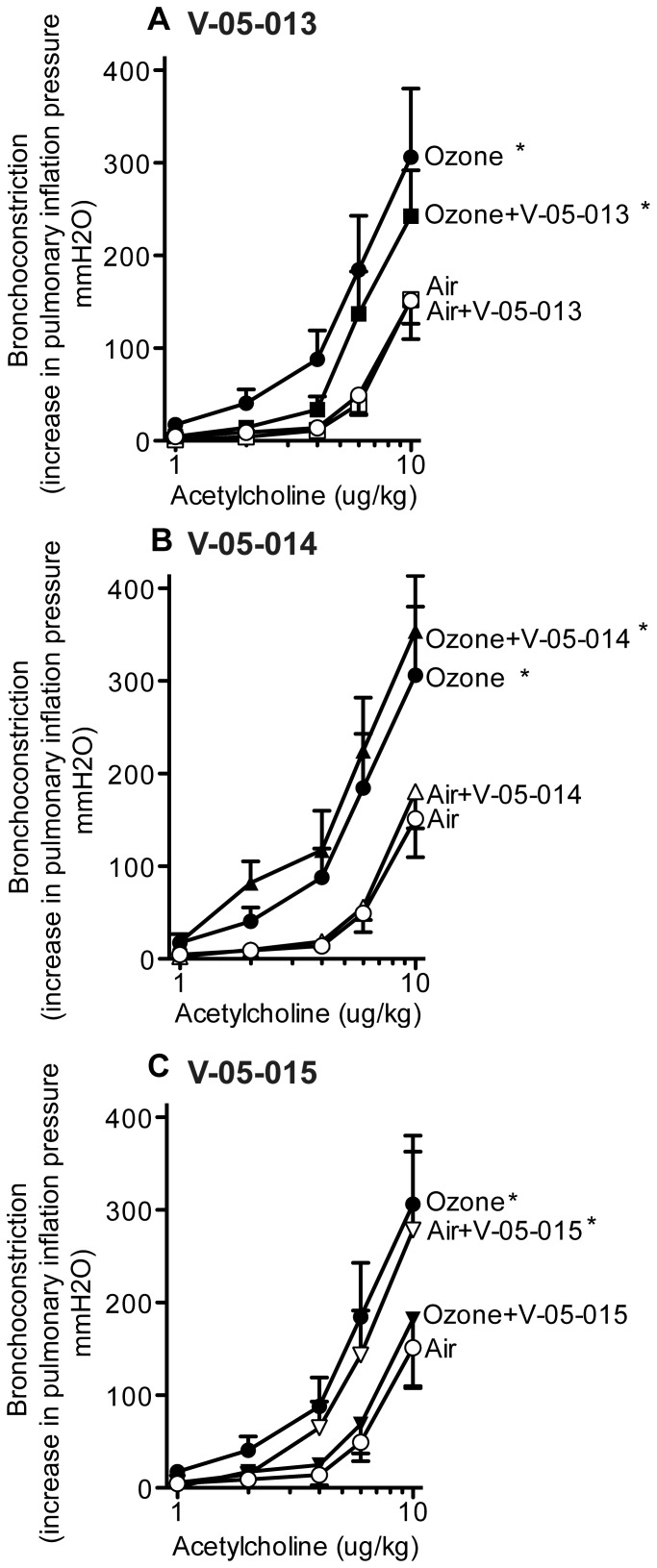
Bronchoconstriction (measured as an increase in inflation pressure in mmH_2_O) in response to intravenous acetylcholine was significantly potentiated one day post-ozone (closed circles) compared to air exposed controls (open circles), and was not blocked by V-05-013 (A closed squares), or V-05-014 (B closed triangles). V-05-015 (C closed inverted triangles) attenuated ozone-induced smooth muscle hyperreactivity while air-exposed animals pretreated with V-05-015 were hyperreactive to intravenous acetylcholine (C open inverted triangles). Ozone and air exposed controls are the same in A-C. *p<0.05, **p<0.01 Significantly different from air exposed controls. Data are mean ± SEM. n = 3–7.

Ozone significantly potentiated bronchoconstriction in response to electrical stimulation of the vagus nerves compared to air-exposed controls as previously reported (Figure 2). Treatment with any of the dual MAPK inhibitors prevented ozone induced airway hyperreactivity (Figures 2A–C). Vehicle treatment had no effect on vagally mediated bronchoconstriction in either air or ozone exposed animals (data not shown). M_2_ muscarinic receptors were dysfunctional in ozone treated animals as gallamine, an M_2_ selective inhibitor, potentiated bronchoconstriction in response to vagal stimulation in air-exposed animals but not in ozone-exposed animals (Figure 3); an effect that is consistent with decreased function of neuronal M_2_ muscarinic receptors [34]. Ozone induced M_2_ receptor dysfunction was prevented by treatment with V-05-014 and V-05-015 (Figure 3B–C), and attenuated by treatment withV-05-013 (Figure 3A). Airway smooth muscle responses to intravenous acetylcholine were potentiated by ozone (Figure 4). This was not prevented by any of the MAPK inhibitors, but was partially attenuated by V-05-015 (Figure 4C). V-05-015 also produced a paradoxical increase in airway response to intravenous acetylcholine in air-exposed animals.

**Figure 5:**
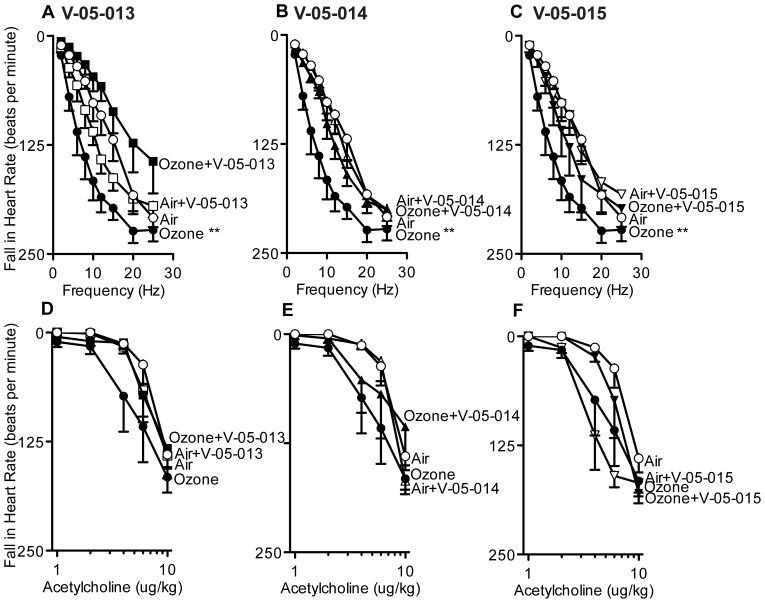
Ozone potentiated vagally mediated falls in heart rate (measured as beats/minute; A–C closed circles) compared to air exposed animals (A–C open circles). Separate pretreatment with all three dual MAPK inhibitors (V-05-013: A closed squares; V-05-014: B closed squares; V-05-015: C closed squares) prevented the ozone-induced potentiation of frequency induced falls in heart rate. Fall in heart rate following intravenous acetylcholine administration was not changed by either ozone, or MAPK inhibitors (D–E). Ozone and air exposed controls are the same for A–C, and are the same for D–F. **p<0.01 Significantly different from air exposed controls. Data are mean ± SEM. n = 3–7.

Ozone exposure potentiated falls in heart rate in response to vagal stimulation compared to air-exposed controls (Figure 5A–C). Ozone and air-exposed controls are the same in figure 5A–C. Separate pretreatment with all three dual MAPK inhibitors prevented the ozone-induced potentiation in falls in heart rate and had no effect in air-exposed animals (Figure 5A–C). Falls in heart rate in response to intravenous acetylcholine were not affected by either ozone or the MAPK inhibitor (Figure 5D–F). Ozone and air exposed controls are the same in figure 5D–F.

### Bronchoalveolar Lavage and Peripheral Blood

**Figure 6:**
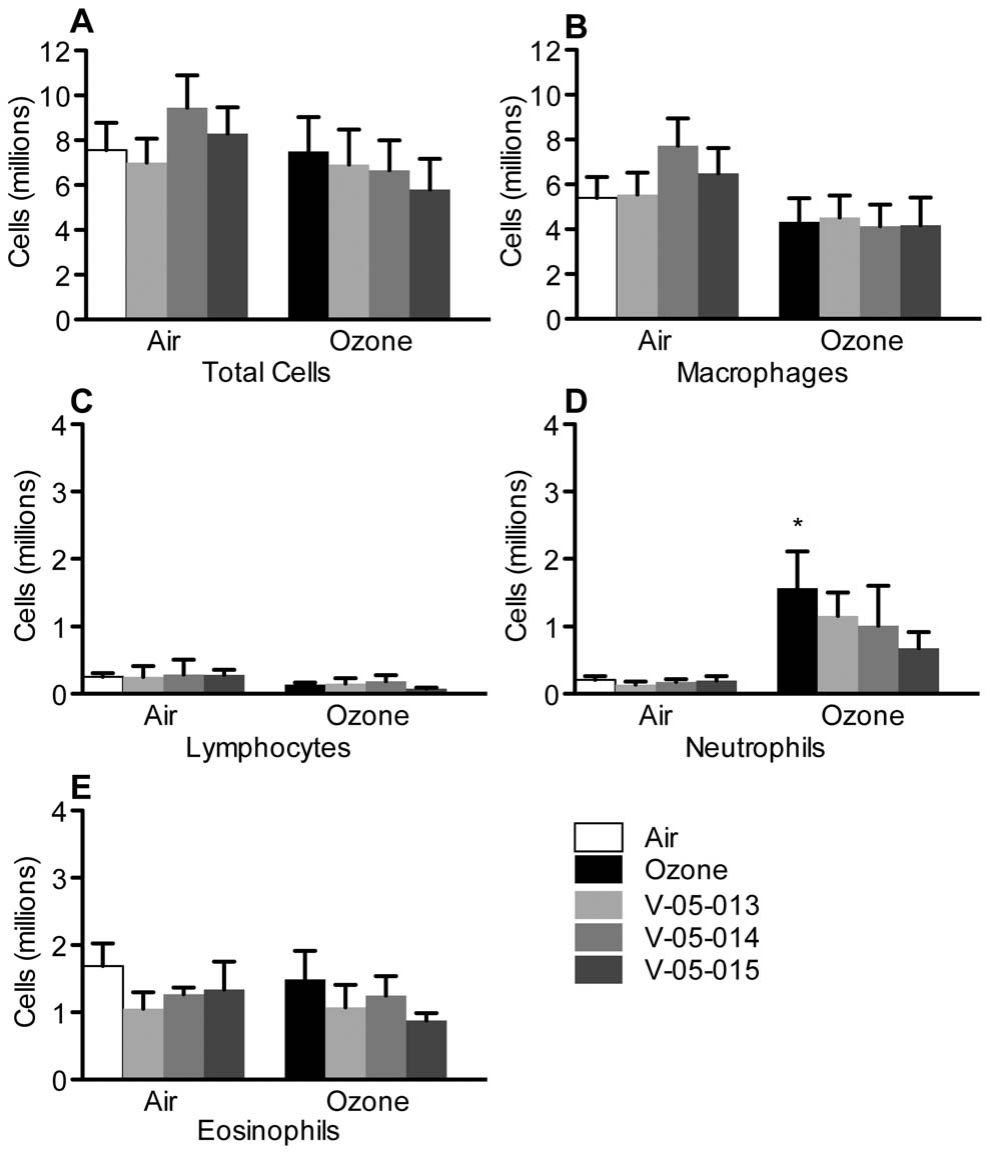
Ozone exposure increased neutrophils in bronchoalveolar lavage (D closed bar). No other inflammatory cell type number was affected by either ozone or the dual p38/JNK MAPK inhibitors. *p<0.05 Significantly different from air exposed controls. Data are mean ± SEM. n = 3–6.

One day after ozone exposure neutrophils were increased in bronchoalveolar lavage (Figure 6D). All the MAPK inhibitors slightly, though not significantly, attenuated the ozone induced increase in neutrophils (Figure 6D). None of the other inflammatory cell types were affected by either ozone or the MAPK inhibitors (Figure 6).

**Figure 7:**
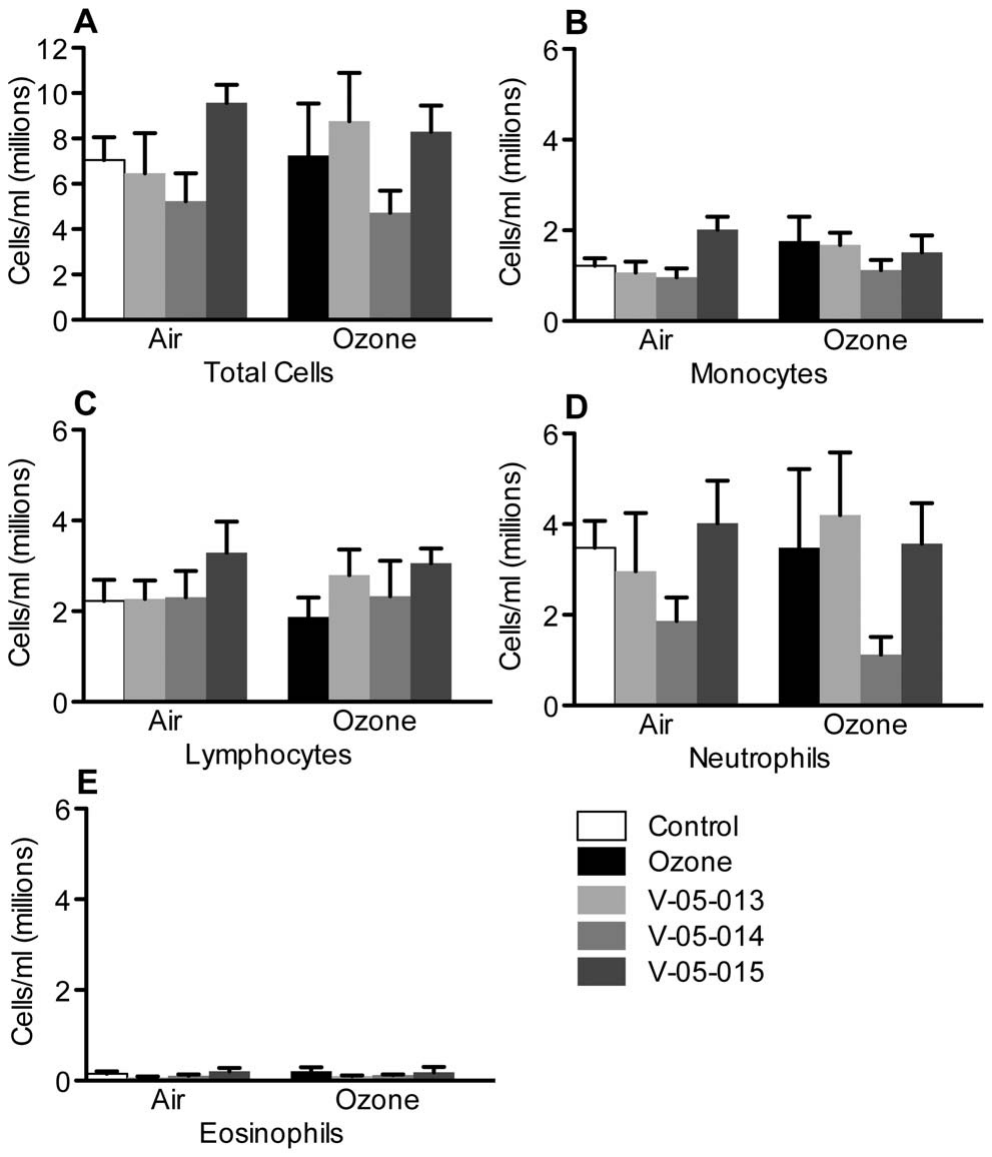
Neither ozone nor the dual p38/JNK MAPK inhibitors affected inflammatory cell numbers in peripheral blood. Data are mean ± SEM. n = 3–6.

There were no significant differences between inflammatory cells in peripheral blood after either ozone exposure, or treatment with the dual MAPK inhibitors (Figure 7).

## Discussion

Ozone induces airway hyperreactivity, measured as potentiation of vagally induced bronchoconstriction, in guinea pigs one day after exposure confirming previous studies [35], [36]. Ozone also significantly potentiated bronchoconstriction in response to intravenous acetylcholine; an effect that has also been previously reported [10]. Blocking both p38 and JNK MAPK with three different, but related, inhibitors prevented vagally mediated hyperreactivity in ozone-exposed animals but had no effect on inflammatory cell numbers in bronchoalveolar lavage. The prevention of vagally mediated hyperreactivity was associated with prevention of ozone induced M_2_ receptor dysfunction that was complete in animals treated with V-05-014 and V-05-015, and partial in animals treated with V-05-013. Ozone induced hyperreactivity to intravenous acetylcholine was partially attenuated by treatment with the MAPK inhibitors.

All three MAPK inhibitors were administered at a dose of 30 mg/kg i.p. one hour before ozone. While the compounds are active with submicromolar potencies, preliminary studies suggested there is a significant shift in *in vivo* potency from the tens of nanomolar to hundreds of nanomolar IC50 s presumably the result of plasma protein binding (unpublished data). Nonetheless, the compounds were chosen because they exhibit adequate pharmacokinetic profiles (table 1) to test our hypothesis *in vivo*. The relatively high *in vivo* clearances and half lives are somewhat limiting, leading to the need for a sufficient dose to demonstrate a role for MAPKs in ozone induced hyperreactivity. However, as with most compounds, different effects could occur at lower doses.

Treatment of air-exposed guinea pigs with any of the three MAPK inhibitors decreased the airway response to vagal stimulation slightly. This effect was most pronounced at high frequency stimulation, but could not be explained by changes in M_2_ receptor function, as the effects of gallamine were not potentiated by the MAPK inhibitors in air exposed animals. This effect was also not due to decreased smooth muscle responsiveness, as the effects of intravenous acetylcholine were not decreased by the MAPK inhibitors. Although response to the MAPK inhibitors was variable in air-exposed animals the overall effect with ozone exposure was prevention of ozone-induced airway hyperreactivity. These minor differences may be due to off target effects of the inhibitors, or to the dose of inhibitors used in this study. Thus, in air exposed guinea pigs, p38 and JNK MAPK inhibitors inhibit vagally induced bronchoconstriction by suppressing release of acetylcholine from airway parasympathetic nerves.

The mechanism for this decreased acetylcholine release is unknown. p38 and JNK are involved in nerve regeneration and development [37], [38] but whether they inhibit ganglionic transmission, action potentials or transmitter release (by a mechanism separate from M_2_ receptors, since there was no change in the response to gallamine) is not well studied. In *Aplysia*, activation of p38 by the peptide neurotransmitter FMRFa leads to long-term depression in sensory neurons in the pleural ganglia [39], although the mechanism is not known. In *Drosophila* motor neurons, expression of constitutively active JNK decreases neurotransmitter release [40] while in primary cultures of rat cortical neurons, IL-1β signaling activates p38, decreasing synaptophysin, a protein involved in synaptic transmission [41]. These varied and sometimes contradictory effects of MAPKs on neural function and transmitter release may be involved in the effects we observed. In neutrophils, activation of p38 MAPK is required for granule exocytosis after stimulation by CXCR1/2 ligands [42]; if neurotransmitter exocytosis were similarly mediated by MAPK, kinase inhibitors would block secretion. Thus, the role of MAPK is cell type dependent and additionally may differ between central neurons where kinases inhibit neurotransmission and peripheral neurons, where they have not been well studied. The data in this paper suggest p38 or JNK MAPK may additionally play a previously unrecognized role in release of acetylcholine from lung parasympathetic nerves.

None of the MAPK inhibitors completely reversed the ozone-induced increase in baseline pulmonary inflation pressure, which is commonly due to airway edema and not increased vagal tone. Ozone also significantly increased the numbers of neutrophils in bronchoalveolar lavage compared to air exposed controls confirming previously published data [35], [36]. However, blocking both p38 and JNK MAPK did not prevent the neutrophil influx. No other inflammatory cell population in the lavage was effected by ozone or by the p38 and JNK MAPK inhibitors. Thus, prevention of ozone-induced airway hyperreactivity did not occur via a decrease in airway inflammatory cells.

Previously we have shown major basic protein, released from eosinophils, inhibits neuronal M_2_ muscarinic receptor function, thereby increasing acetylcholine release and subsequently leading to increased bronchoconstriction and airway hyperreactivity after ozone exposure [43], [44]. Depletion of eosinophils with an antibody to IL-5, or blocking major basic protein with heparin, prevents M_2_ receptor dysfunction and ozone-induced airway hyperreactivity one day post-ozone exposure [11]. Thus, although neutrophils are the cells that increase in the bronchoalveolar lavage after ozone, it is tissue eosinophils around airway nerves that mediate ozone-induced hyperreactivity. In eosinophils, eotaxin and IL-5 signal through both ERK and p38 MAPK activation [45], [46]. Inhibition of p38 reduces eosinophil degranulation as measured by decreased eosinophil cationic protein release [45]. Major basic protein has also been shown to alter smooth muscle contractility [47]. Thus, while not tested directly in this study, blocking eosinophil degranulation with MAPK inhibitors could also contribute to preventing smooth muscle hyperreactivity.

Thus, p38 and JNK MAPK inhibitors inhibit ozone-induced hyperreactivity by multiple mechanisms. Exposure to high levels of environmental ozone increases hospitalizations from asthma exacerbations. Over 4 million children and 10 million adults with asthma live in counties with unhealthy levels of ozone, and those with asthma are an especially susceptible population to the adverse health effects of ozone [1]. Our data show that treatment with p38 and JNK inhibitors, immediately prior to ozone exposure prevented subsequent development of airway hyperreactivity. Currently there is no specific therapy for ozone related asthma complications and our data suggest both p38 and JNK are potential targets for additional therapeutic candidates; and that inhibitors could be tested as prophylactic treatment for asthma exacerbations on days with anticipated high ozone.
